# Coding-complete genome sequence of a measles virus genotype B3 isolate from Rabat, Morocco, in 2024

**DOI:** 10.1128/mra.00836-24

**Published:** 2025-05-05

**Authors:** Safae Elkochri, Idrissa Diawara, Zineb Rhazzar, El Mehdi Belouad, Youssef El kadiri, Maryam Benlamari, Taha Chouati, Boutaina Elgharbaoui, Narjisse Ahmadi, Amine Idriss Lahlou, Nadia Touil, Khalid Ennibi, Boutayeb Saber, Lahcen Belyamani, Elmostafa El Fahime

**Affiliations:** 1 Center of Virology Infectious and Tropical Diseases, the Mohammed V Military Training Hospital, Rabat, Morocco; 2 Department of Microbiology, Faculty of Medicine & Pharmacy, Hassan II University of Casablanca 92957 , Casablanca, Morocco; 3 Mohammed VI University of Sciences and Health (UM6SS), Casablanca, Morocco; 4 Mohammed VI Center for Research & Innovation (CM6RI), Rabat, Morocco; 5 Immunopathology Research Team (ERIP), Faculty of Medicine & Pharmacy, University Mohammed V 97980, Rabat, Rabat-Sale-Zemmour-Zaer, Morocco; 6 Molecular Biology and Functional Genomics Platform, National Center For Scientific and Technical Research 204579, Rabat, Rabat-Sale-Zemmour-Zaer, Morocco; Katholieke Universiteit Leuven, Leuven, Belgium

**Keywords:** measles virus, genotype, Sanger sequencing, coding whome genome

## Abstract

We determined the coding-complete genome sequence of a measles virus that was isolated in Rabat from a Moroccan patient on 23 March 2024. Phylogenetic analysis showed that this virus, named MVi/Rabat.MAR/29.24, belongs to lineage B3 and is closely related to a USA isolate.

## ANNOUNCEMENT

The Ministry of Health and Social Protection issued a warning on March 2024, regarding the surge of measles cases nationwide (1). This disease is caused by the measles virus (MV), the prototype of the *Morbilliviru*s genus and *Paramyxoviridae* family (2). The MV is an enveloped and single -stranded, negative sense RNA virus. The WHO currently recognizes eight clades, designated A–H and 24 genotypes (or subtypes) (3). MV genomes are generally composed of 15,894 nt and encode six structural proteins (N, P, M, F, H, and L) and two nonstructural proteins (V, C)

We performed genome analysis of a circulating MV virus to help trace the spread of MV during the recent epidemics.

MV virus was isolated from a nasopharyngeal swab of a 32-year-old female admitted with fever, cough, and a red blotchy rash without serious complications at the Military Training Hospital, Rabat, on 23 March 2024.

The virus was isolated in Vero.DogSLAM as described previously (1). RNA was extracted from infected cells using the NucleoSpin RNA Virus Isolation Kit (Macherey-Nagel, Germany) and quantified using NanoDrop Spectrophotometer (Thermo Fisher Scientific, USA). cDNA was prepared with 200 ng of RNA and random hexamer using the RevertAid RT Kit (Thermo Fisher Scientific, USA).

Published (4) and custom-designed primers covering the whole MV reference genomes (NC_001498.1 or MN630023.1) were used with MyFi Mix (Meridains Bioscience) for PCR amplification at *T*_*m*_ 55°C (Table 1). Twenty-one amplicons were sequenced in both directions (42 reads in total) on an Applied Biosystems. We used defaults parameters of Unipro Ugene version 50.5 (5) to assemble the nucleotide sequences aligned with the RefSeq (NC_001498.1).

**TABLE 1:** 

Primer name	Sequence (5′ to 3′)	Amplicon size (bp)	Reference
UTR5-F	ACCAAACAAAGTTGGGTAAGGAT	541	This study
MVR-1R	CCACCCGAACCTGGATTGATC		This study
AF1-2	CGATCCAGACTTCTGGACCG	1,098	Namuwulya et al. (4)
AR1-2	TCTGGGTCCAACTGCTCTGC		This study
MVR-2F	GAACTCGGTATCACTGCCGAG	877	This study
MVR-2R	CTGTAACCCAGCGCTTGAT		This study
BF2_5	GCACCTCGCATCCGC	604	Namuwulya et al. (4
AR1_4	CACACTCGGGGACATTCCC		Namuwulya et al. (4)
MVR-3F	GCAACAACTTTCTGAAGCCTGG	954	This study
MVR-3R	TGGGGTTGGCAGGTAAGTTG		This study
MVR-4F	AGCCAACGATCTTGCCAAGT	985	This study
MVR-4R	CGAGTTGTGCATGGAGGGT		This study
AF3_10	TCACATCGGGAACTTCAGGAGA	579	Namuwulya et al. (4)
BR2_8	GGTTGGTCCTCGGGGGT		Namuwulya et al. (4)
MVR-5F	GTTCCTCAAGAATTCCGCATTTAC	1,047	This study
MVR-5R	GTCTCCTTTAGGGGTGGGGAG		This study
MVR-6F	AACCGCAAAGGACATCAGCAT	1,002	This study
MVR-6R	CTATGAAGTAGGACTCTGTGTCGA		This study
BR4-13	TTTATCCGGTCTCGTTGCGG	1,046	This study
BF4_13	TAAAGGCTCGGATAACTCACGTC		Namuwulya et al. (4)
MVR-7F	GCTTCCCTCTAGCCGAACAA	1,149	This study
MVR-7R	CTTGATTGTCAGCGATGACACC		This study
AF5_17	AAGCTGGGTGTCTGGAAATCC	1,099	Namuwulya et al. (4)
AR5-17	GAGTCGAGCATACTCCAGGATG		This study
MVR-8 F	GGACTCGCTATCTGTCAACCAG	1,078	This study
MVR-8R	TCTGGGGTGGCCGAAACT		This study
BF6_21	TTAATGACAGAGACCGCTATGACC	766	Namuwulya et al. (4)
AR5_20	CGGTTCCCTTGGGAGGG		Namuwulya et al. (4)
MVR-9F	AGTTTACCCGAAAGAGTTCCTGC	818	This study
MVR-9R	TGCTGATGGTCCACAGCTTCT		This study
MVR-10F	CGCCCATATCCCGTTATGCA	816	This study
MVR-10R	GAGTCTCCTGGTTGTTGTGTCA		This study
AF7_26	CTCGCCTCGCTAATGCCT	534	Namuwulya et al. (4)
BR6_23	CGCCCACATATGGCTTCTTAGA		Namuwulya et al. (4)
MVR-11F	CAATTCAGGGCAGGGATGGT	1,217	This study
MVR-11R	GACAACAGCTCACCCATCTGA		This study
BF8-29	TTTCAGCTCTCATAGGGGATGATG	844	Namuwulya et al. (4)
AR7-28	GGAAGATTGTGCTTGCTTGTGT		Namuwulya et al. (4)
BF8-31	ATCAAGGATTTCAGACCCCCAC	1,370	This study
BR8_31	CATTCTTTGGTCTCCTTGACTGTT		Namuwulya et al. (4)
UTR3-F	CCGTATTGGTAAGTAGTAGGCA	476	This study
UTR3-R	ACCAGACAAAGCTGGGAATAGA		This study

**Fig 1:**
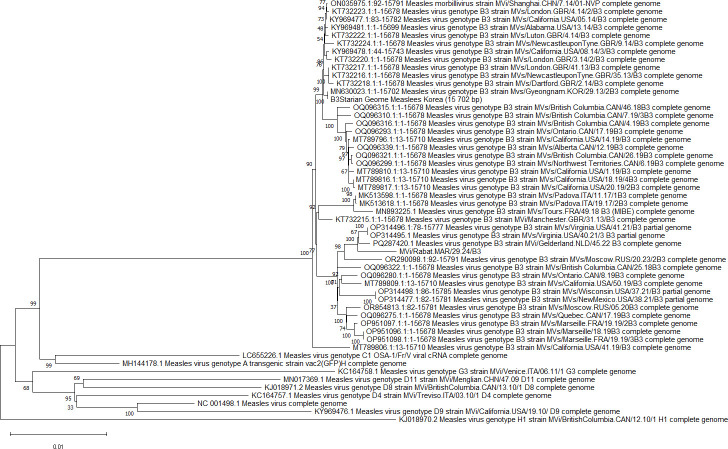
A phylogenetic tree representing the coding-complete genome sequence of the Measles strain MVi/Rabat.MAR/29.24/[B3] along with 51 other RefSeq genomes selected from different regions at the nucleotide level. The evolutionary history was inferred using the neighbor-joining method to generate the tree. The percentage of replicate trees in which the associated taxa clustered together during the bootstrap test (500 replicates) is indicated next to the branches. The tree is drawn to scale, with branch lengths in the same units as the evolutionary distances used to infer the phylogenetic tree. These evolutionary distances were computed using the maximum composite likelihood method and are expressed in the number of base substitutions per site. This analysis involved 51 nucleotide sequences, and all ambiguous positions were removed.

The assembled genome consists of 15,896 nucleotides, and has an identity match of 99.50% and 99.44%, respectively, with the MVs/Virginia.USA/40.21/3[B3] (Gi: OP314495.1) and MVi/Gelderland.NLD/45.22[B3] (Gi:PQ287420.1) (Fig. 1).

Our strain is classified as a B3 genotype as per the WHO genotyping approach (6) with 99.50% similarity to MVs/Virginia.USA/40.21/3[B3] strain.

## Data Availability

The coding-complete genome sequence of the MVi/Rabat.MAR/29.24/[B3] isolate is available at GenBank under the accession number Gi: PQ069064.2.
